# Supplementary material for: Quantifying the association of natal household wealth with women’s early marriage in Nepal
Source: PeerJ. 2021 Dec 16;9:e12324. doi: 10.7717/peerj.12324 (PMC8684741; doi:10.7717/peerj.12324)
Supplement: Supplemental Information 1 [file peerj-09-12324-s001.docx]

**Table S1. Differences in traits between women measured in natal vs marital home**

|  | **1: Assets measured in natal household (*n*=2,432)** | | **2: Assets measured in marital household**  **(*n*=14,103)** | | ***p-*value^1^** |
| --- | --- | --- | --- | --- | --- |
|  | **Median** | **IQR** | **Median** | **IQR** |  |
| Women’s age (y) | 21 | 4 | 22 | 5 | <0.001 |
| Women’s age at marriage (y)^3^ | 15 | 3 | 15 | 3 | na |
| Time since marriage (y)^3^ | 5 | 5 | 7 | 6 | <0.001 |
| Women’s age at first pregnancy (y)^4^ | 18 | 3 | 18 | 3 | na |
|  | **Frequency** | **%** | **Frequency** | **%** | ***p-*value^2^** |
| Trial arm |  |  |  |  | <0.001 |
| Control | 429 | 17.6 | 3,371 | 23.9 |  |
| Women’s Group | 446 | 18.3 | 3,353 | 23.8 |  |
| Women’s Group with cash transfer | 793 | 32.6 | 3,889 | 27.6 |  |
| Women’s Group with food supplement | 764 | 31.4 | 3,490 | 24.7 |  |
| Women’s age at marriage (y)^3^ |  |  |  |  | <0.001 |
| <14 years | 666 | 27.4 | 5,038 | 35.7 |  |
| 15 years | 574 | 23.6 | 3,420 | 24.3 |  |
| 16 years | 356 | 14.6 | 1,891 | 13.4 |  |
| 17 years | 408 | 16.8 | 1,901 | 13.5 |  |
| ≥18 years | 428 | 17.6 | 1,851 | 13.1 |  |
| Women’s education level (y)^3^ |  |  |  |  | 0.216 |
| None | 1,610 | 66.2 | 9,571 | 67.9 |  |
| Primary (1-5 years) | 244 | 10.0 | 1,434 | 10.2 |  |
| Lower-secondary (6-8 years) | 196 | 8.1 | 1,096 | 7.8 |  |
| Secondary or higher (≥9 years) | 382 | 15.7 | 2,000 | 14.2 |  |
| Household asset score^5^ |  |  |  |  |  |
| 1: poorest | 525 | 21.6 | 2,902 | 20.6 | 0.156 |
| 2: 2^nd^ poorest | 484 | 19.9 | 2,841 | 20.2 |  |
| 3: mid | 528 | 21.7 | 2,879 | 20.4 |  |
| 4: 2^nd^ richest | 465 | 19.1 | 2,711 | 19.2 |  |
| 5: richest | 430 | 17.7 | 2,761 | 19.6 |  |
| Agrarian land-holding^6^ |  |  |  |  | 0.013 |
| None | 951 | 39.1 | 5,042 | 35.8 |  |
| 0.01 to 0.5 hectares | 750 | 30.8 | 4,557 | 32.3 |  |
| 0.51 to 0.99 hectares | 344 | 14.1 | 2,037 | 14.4 |  |
| ≥1 hectare | 387 | 15.9 | 2,462 | 17.5 |  |
| Access to big bazaar^7^ |  |  |  |  | 0.006 |
| <30 minutes | 798 | 32.8 | 4,971 | 35.3 |  |
| 30-59 minutes | 934 | 38.4 | 4,949 | 35.1 |  |
| 60-89 minutes | 472 | 19.4 | 2,926 | 20.8 |  |
| >90 minutes | 228 | 9.4 | 1,250 | 8.9 |  |
| Caste |  |  |  |  | <0.001 |
| Disadvantaged: Dalit | 494 | 20.3 | 2,624 | 18.6 |  |
| Disadvantaged: Muslim | 468 | 19.2 | 2,270 | 16.1 |  |
| Middle: Janjati, Terai castes | 927 | 38.1 | 6,024 | 42.7 |  |
| Advantaged: Yadav, Brahmin | 543 | 22.3 | 3,185 | 22.6 |  |

IQR, interquartile range. ^1^Kruskal-Wallis test. ^2^Chi-squared test. ^3^*n*=14,101 in marital household. ^4^*n*=2,431 in natal household. ^5^*n*=14,094 in marital household. ^6^*n*=14,098 in marital household. ^7^*n*=14,096 in marital household. *n*=14,102 in marital household.
