# Supplementary material for: Quantifying the association of natal household wealth with women’s early marriage in Nepal
Source: PeerJ. 2021 Dec 16;9:e12324. doi: 10.7717/peerj.12324 (PMC8684741; doi:10.7717/peerj.12324)
Supplement: Supplemental Information 2 [file peerj-09-12324-s002.docx]

**Table S2. Differences in individual assets by natal household asset quintiles (*n*=2,432)**

|  | **Natal household asset quintiles** | | | | | | | | | |  |
| --- | --- | --- | --- | --- | --- | --- | --- | --- | --- | --- | --- |
|  | **Poorest** | | **2^nd^ Poor** | | **Mid** | | **2^nd^ Rich** | | **Richest** | |  |
| **Individual assets** | **Mean** | **SD** | **Mean** | **SD** | **Mean** | **SD** | **Mean** | **SD** | **Mean** | **SD** | ***p-value^1^*** |
| Number of rooms used for sleeping in home | 1.9 | 0.6 | 2.4 | 0.8 | 2.9 | 0.8 | 3.1 | 0.8 | 3.5 | 0.7 | <0.001 |
|  | **Frequency** | **%** | **Frequency** | **%** | **Frequency** | **%** | **Frequency** | **%** | **Frequency** | **%** | ***p-value^2^*** |
| **Wall materials** |  |  |  |  |  |  |  |  |  |  | <0.001 |
| Mud & stems, straw, wooden planks, metal sheets | 552 | 99.4 | 466 | 96.3 | 448 | 84.8 | 195 | 41.9 | 4 | 0.9 |  |
| Cement & stems, mud & ricks, stones, other | 2 | 0.4 | 13 | 2.7 | 37 | 7.0 | 51 | 11.0 | 37 | 8.6 |  |
| Cement & brick | 1 | 0.2 | 5 | 1.0 | 43 | 8.1 | 219 | 47.1 | 389 | 90.5 |  |
| **Roofing materials** |  |  |  |  |  |  |  |  |  |  | <0.001 |
| Thatch | 275 | 52.4 | 38 | 7.9 | 4 | 0.8 | 1 | 0.2 | 0 | 0.0 |  |
| Traditional tiles (Kapada) | 226 | 43.0 | 335 | 69.2 | 309 | 58.5 | 252 | 54.2 | 40 | 9.3 |  |
| Tiles, asbestos, metal, other | 24 | 4.6 | 110 | 22.7 | 211 | 40.0 | 137 | 29.5 | 38 | 8.8 |  |
| Cement | 0 | 0.0 | 1 | 0.2 | 4 | 0.8 | 75 | 16.1 | 352 | 81.9 |  |
| **Flooring materials** |  |  |  |  |  |  |  |  |  |  | <0.001 |
| Dirt, dung | 525 | 100.0 | 484 | 100.0 | 527 | 99.8 | 450 | 96.8 | 147 | 34.2 |  |
| Cement or other non-dirt | 0 | 0.0 | 0 | 0.0 | 1 | 0.2 | 15 | 3.2 | 283 | 65.8 |  |
| **Toilet facilities** |  |  |  |  |  |  |  |  |  |  | <0.001 |
| Bush, stream, open areas | 522 | 99.4 | 465 | 96.1 | 460 | 87.1 | 215 | 46.2 | 67 | 15.6 |  |
| Pit, other | 1 | 0.2 | 3 | 0.6 | 19 | 3.6 | 37 | 8.0 | 20 | 4.7 |  |
| Pan | 2 | 0.4 | 16 | 3.3 | 49 | 9.3 | 200 | 43.0 | 324 | 75.3 |  |
| Flush | 0 | 0.0 | 0 | 0.0 | 0 | 0.0 | 13 | 2.8 | 19 | 4.4 |  |
| **Access to electricity** |  |  |  |  |  |  |  |  |  |  |  |
| No access | 147 | 28.0 | 37 | 7.6 | 11 | 2.1 | 13 | 2.8 | 5 | 1.2 | <0.001 |
| Hooking | 173 | 33.0 | 75 | 15.5 | 38 | 7.2 | 21 | 4.5 | 13 | 3.0 |  |
| Solar panel | 20 | 3.8 | 14 | 2.9 | 9 | 1.7 | 2 | 0.4 | 1 | 0.2 |  |
| Own, neighbour | 185 | 35.2 | 358 | 74.0 | 470 | 89.0 | 429 | 92.3 | 411 | 95.6 |  |
| **Water source** |  |  |  |  |  |  |  |  |  |  |  |
| Public, neighbours | 338 | 64.4 | 140 | 28.9 | 56 | 10.6 | 45 | 9.7 | 14 | 3.3 | <0.001 |
| Own yard | 76 | 14.5 | 148 | 30.6 | 168 | 31.8 | 142 | 30.5 | 115 | 26.7 |  |
| Inside, deep borehole | 111 | 21.1 | 196 | 40.5 | 304 | 57.6 | 278 | 59.8 | 301 | 70.0 |  |
| **Non-biomass fuel use** |  |  |  |  |  |  |  |  |  |  |  |
| Biomass burner | 525 | 100.0 | 483 | 99.8 | 524 | 99.2 | 440 | 94.6 | 374 | 87.0 | <0.001 |
| Non-biomass burner | 0 | 0.0 | 1 | 0.2 | 4 | 0.8 | 25 | 5.4 | 56 | 13.0 |  |

SD, Standard Deviation. ^1^ANOVA. ^2^Chi-Squared test.
