# Supplementary material for: Quantifying the association of natal household wealth with women’s early marriage in Nepal
Source: PeerJ. 2021 Dec 16;9:e12324. doi: 10.7717/peerj.12324 (PMC8684741; doi:10.7717/peerj.12324)
Supplement: Supplemental Information 3 [file peerj-09-12324-s003.docx]

**Table S3. Associations of natal household asset score with women’s early marriage in the full sample of women aged 12-39 years**

|  | **Hypothesis 1** | | | | | | | |
| --- | --- | --- | --- | --- | --- | --- | --- | --- |
|  | **Model 1: Marrying <15 years**  *n*=1,396^1^ *R*^2^ =0.098 | | **Model 2: Marrying <16 years**  *n*=2,279^2^ *R*^2^ =0.080 | | **Model 3: Marrying <17 years**  *n*=2,882^3^ *R*^2^ =0.084 | | **Model 4: Marrying <18 years**  *n*=3,379^4^ *R*^2^ =0.074 | |
|  | **aOR (95% CI)** | ***p-*value** | **aOR (95% CI)** | ***p-*value** | **aOR (95% CI)** | ***p-*value** | **aOR (95% CI)** | ***p-*value** |
| Women’s age (y) | 0.88 (0.84, 0.91) | <0.001 | 0.89 (0.86, 0.91) | <0.001 | 0.88 (0.85, 0.90) | <0.001 | 0.88 (0.86, 0.90) | <0.001 |
| Asset score |  |  |  |  |  |  |  |  |
| Poorest | 2.90 (1.91, 4.41) | <0.001 | 2.40 (1.67, 3.45) | <0.001 | 2.25 (1.59, 3.18) | 0.001 | 2.00 (1.43, 2.80) | <0.001 |
| 2^nd^ poorest | 2.20 (1.45, 3.33) | <0.001 | 1.70 (1.19, 2.43) | 0.004 | 1.66 (1.18, 2.32) | 0.003 | 1.50 (1.08, 2.08) | 0.015 |
| Mid | 2.04 (1.35, 3.08) | 0.001 | 1.80 (1.26, 2.56) | 0.001 | 1.70 (1.21, 2.37) | 0.002 | 1.57 (1.14, 2.17) | 0.006 |
| 2^nd^ richest | 2.16 (1.41, 3.30) | <0.001 | 1.83 (1.27, 2.65) | 0.001 | 1.80 (1.27, 2.56) | 0.001 | 1.69 (1.20, 2.37) | 0.003 |
| Richest (ref) | 1.00 |  | 1.00 |  | 1.00 |  | 1.00 |  |
| Intercept | 19.65 (7.24, 53.33) | <0.001 | 36.68 (15.75, 85.46) | <0.001 | 65.95 (29.99, 145.02) | <0.001 | 76.50 (36.33, 161.08) | 0.001 |

Models include fixed and random effects estimates for geographic clusters and control for trial arm. As associations of trial arm with early marriage across the age groupings were not statistically significant, they are not reported in Tables. aOR, adjusted Odds Ratio. CI, 95% Confidence Interval. ^1^*n*=428 married ≥18y vs *n*=968 married <15y. ^2^*n*=428 married ≥18y vs *n*=1,851 married <16y. ^3^*n*=428 married ≥18y vs *n*=2,454 married <17y. ^4^*n*=428 married ≥18y vs *n*=2,951 married <18y.
