# Supplementary material for: Quantifying the association of natal household wealth with women’s early marriage in Nepal
Source: PeerJ. 2021 Dec 16;9:e12324. doi: 10.7717/peerj.12324 (PMC8684741; doi:10.7717/peerj.12324)
Supplement: Supplemental Information 4 [file peerj-09-12324-s004.docx]

**Table S4. Broader socio-economic factors associated with women’s early marriage in the full sample of women aged 12-39 years**

| **Hypothesis 2** | | | | | | | | |
| --- | --- | --- | --- | --- | --- | --- | --- | --- |
|  | **Model 1: Marrying <15 years**  *n*=1,396^1^ *R*^2^ =0.237 | | **Model 2: Marrying <16 years**  *n*=2,279^2^ *R*^2^ =0.192 | | **Model 3: Marrying <17 years**  *n*=2,882^3^ *R*^2^ =0.174 | | **Model 4: Marrying <18 years**  *n*=3,379^4^ *R*^2^ =0.153 | |
|  | **aOR (95% CI)** | ***p-*value** | **aOR (95% CI)** | ***p-*value** | **aOR (95% CI)** | ***p-*value** | **aOR (95% CI)** | ***p-*value** |
| Women’s age (y) | 0.85 (0.81, 0.88) | <0.001 | 0.86 (0.83, 0.89) | <0.001 | 0.85 (0.82, 0.88) | <0.001 | 0.85 (0.83, 0.88) | <0.001 |
| Asset score |  |  |  |  |  |  |  |  |
| Poorest | 1.06 (0.62, 1.81) | 0.845 | 1.02 (0.65, 1.59) | 0.941 | 1.08 (0.71, 1.65) | 0.710 | 1.00 (0.67, 1.50) | 0.981 |
| 2^nd^ poorest | 0.98 (0.59, 1.61) | 0.923 | 0.82 (0.54, 1.24) | 0.345 | 0.93 (0.63, 1.36) | 0.706 | 0.88 (0.61, 1.27) | 0.503 |
| Mid | 0.96 (0.59, 1.56) | 0.863 | 0.95 (0.63, 1.43) | 0.805 | 1.02 (0.70, 1.49) | 0.907 | 0.98 (0.69, 1.40) | 0.924 |
| 2^nd^ richest | 1.48 (0.91, 2.42) | 0.113 | 1.32 (0.88, 1.99) | 0.182 | 1.41 (0.97, 2.06) | 0.074 | 1.30 (0.91, 1.87) | 0.151 |
| Richest (ref) | 1.00 |  | 1.00 |  | 1.00 |  | 1.00 |  |
| Women’s education |  |  |  |  |  |  |  |  |
| None | 13.49 (8.40, 21.67) | <0.001 | 9.33 (6.45, 13.50) | <0.001 | 6.76 (4.84, 9.45) | <0.001 | 5.29 (3.86, 7.25) | <0.001 |
| Primary (1-5y) | 8.48 (4.77, 15.08) | <0.001 | 6.13 (3.83, 9.82) | <0.001 | 4.49 (2.90, 6.95) | <0.001 | 3.73 (2.45, 5.67) | <0.001 |
| Lower-secondary (6-8y) | 4.99 (2.77, 9.01) | <0.001 | 4.78 (2.94, 7.77) | <0.001 | 4.30 (2.75, 6.73) | <0.001 | 3.73 (2.42, 5.73) | <0.001 |
| Secondary/higher (≥9) (ref) | 1.00 |  | 1.00 |  | 1.00 |  | 1.00 |  |
| Agrarian land |  |  |  |  |  |  |  |  |
| None | 1.06 (0.64, 1.75) | 0.815 | 1.05 (0.69, 1.59) | 0.836 | 0.96 (0.65, 1.42) | 0.854 | 0.98 (0.68, 1.43) | 0.935 |
| 0.01 to 0.5 hectares | 1.30 (0.81, 2.08) | 0.282 | 1.41 (0.95, 2.10) | 0.086 | 1.30 (0.90, 1.88) | 0.157 | 1.31 (0.93, 1.86) | 0.127 |
| 0.51 to 0.99 hectares | 1.01 (0.59, 1.68) | 0.989 | 1.12 (0.73, 1.72) | 0.598 | 1.11 (0.75, 1.65) | 0.593 | 1.13 (0.78, 1.64) | 0.528 |
| ≥1 hectare (ref) | 1.00 |  | 1.00 |  | 1.00 |  | 1.00 |  |
| Access to big bazaar |  |  |  |  |  |  |  |  |
| <30 min (ref) | 1.00 |  | 1.00 |  | 1.00 |  | 1.00 |  |
| 30-59 minutes | 1.03 (0.69, 1.56) | 0.871 | 1.11 (0.79, 1.55) | 0.561 | 0.99 (0.72, 1.36) | 0.938 | 1.02 (0.75, 1.38) | 0.921 |
| 60-89 minutes | 1.03 (0.63, 1.68) | 0.915 | 1.14 (0.76, 1.70) | 0.540 | 0.97 (0.66, 1.41) | 0.862 | 0.97 (0.68, 1.39) | 0.877 |
| ≥90 minutes | 1.20 (0.65, 2.23) | 0.562 | 1.14 (0.67, 1.96) | 0.622 | 0.91 (0.55, 1.52) | 0.730 | 1.02 (0.63, 1.66) | 0.933 |
| Caste |  |  |  |  |  |  |  |  |
| Disadvantaged: Dalit | 0.96 (0.56, 1.61) | 0.864 | 1.12 (0.72, 1.73) | 0.620 | 1.16 (0.77, 1.75) | 0.486 | 1.18 (0.79, 1.75) | 0.414 |
| Disadvantaged: Muslim | 1.08 (0.66, 1.79) | 0.756 | 1.12 (0.72, 1.73) | 0.617 | 1.09 (0.73, 1.64) | 0.677 | 1.08 (0.73, 1.59) | 0.716 |
| Middle: Janjati, Terai castes | 1.01 (0.67, 1.53) | 0.960 | 1.13 (0.80, 1.60) | 0.473 | 1.11 (0.81, 1.52) | 0.530 | 1.08 (0.80, 1.46) | 0.630 |
| Advantaged: Yadav, Brahmin (ref) | 1.00 |  | 1.00 |  | 1.00 |  | 1.00 |  |
| Intercept | 8.06 (2.46, 26.38) | 0.001 | 16.44 (6.14, 44.02) | <0.001 | 39.16 (15.80, 97.05) | <0.001 | 51.78 (22.10, 121.30) | <0.001 |

Models include fixed and random effects estimates for geographic clusters and control for trial arm. aOR, adjusted Odds Ratio. CI, 95% Confidence Interval. ^1^*n*=428 married ≥18y vs *n*=968 married <15y. ^2^*n*=428 married ≥18y vs *n*=1,851 married <16y. ^3^*n*=428 married ≥18y vs *n*=2,454 married <17y. ^4^*n*=428 married ≥18y vs *n*=2,951 married <18y.
