# Supplementary material for: Quantifying the association of natal household wealth with women’s early marriage in Nepal
Source: PeerJ. 2021 Dec 16;9:e12324. doi: 10.7717/peerj.12324 (PMC8684741; doi:10.7717/peerj.12324)
Supplement: Supplemental Information 5 [file peerj-09-12324-s005.docx]

**Table S5. Associations of natal household asset score and women’s early marriage in the full sample of uneducated women aged 12-39 years**

|  | **Hypothesis 3** | | | | | | | |
| --- | --- | --- | --- | --- | --- | --- | --- | --- |
|  | **Model 1: Marrying <15 years**  *n*=914^1^ *R*^2^ =0.096 | | **Model 2: Marrying <16 years**  *n*=1,499^2^ *R*^2^ =0.089 | | **Model 3: Marrying <17 years**  *n*=1,841^3^ *R*^2^ =0.091 | | **Model 4: Marrying <18 years**  *n*=2,116^4^ *R*^2^ =0.087 | |
|  | **aOR (95% CI)** | ***p-*value** | **aOR (95% CI)** | ***p-*value** | **aOR (95% CI)** | ***p-*value** | **aOR (95% CI)** | ***p-*value** |
| Women’s age (y) | 0.90 (0.86, 0.94) | <0.001 | 0.90 (0.87, 0.94) | <0.001 | 0.89 (0.86, 0.93) | <0.001 | 0.90 (0.86, 0.93) | <0.001 |
| Asset score |  |  |  |  |  |  |  |  |
| Poorest | 0.98 (0.51, 1.86) | 0.944 | 0.88 (0.49 1.57) | 0.655 | 0.91 (0.52, 1.59) | 0.746 | 0.83 (0.48, 1.43) | 0.509 |
| 2^nd^ poorest | 0.83 (0.43, 1.62) | 0.593 | 0.71 (0.39, 1.29) | 0.257 | 0.74 (0.42, 1.32) | 0.308 | 0.70 (0.40, 1.22) | 0.208 |
| Mid | 0.94 (0.48, 1.83) | 0.848 | 0.84 (0.46, 1.54) | 0.574 | 0.83 (0.47, 1.49) | 0.540 | 0.79 (0.45, 1.39) | 0.412 |
| 2^nd^ richest | 1.73 (0.81, 3.70) | 0.154 | 1.45 (0.72, 2.90) | 0.297 | 1.43 (0.73, 2.81) | 0.295 | 1.42 (0.73, 2.74) | 0.301 |
| Richest (ref) | 1.00 |  | 1.00 |  | 1.00 |  | 1.00 |  |
| Intercept | 47.07 (11.84, 187.11) | <0.001 | 81.80 (23.61, 283.42) | <0.001 | 128.66 (39.82, 415.64) | <0.001 | 142.91 (46.04, 443.62) | <0.001 |

Models include fixed and random effects estimates for geographic clusters and control for trial arm. aOR, adjusted Odds Ratio. CI, 95% Confidence Interval. ^1^*n*=206 married ≥18y vs *n*=708 married <15y. ^2^*n*=206 married ≥18y vs *n*=1,293 married <16y. ^3^*n*=206 married ≥18y vs *n*=1,635 married <17y. ^4^*n*=206 married ≥18y vs *n*=1,910 married <18y.
