# Supplementary material for: Quantifying the association of natal household wealth with women’s early marriage in Nepal
Source: PeerJ. 2021 Dec 16;9:e12324. doi: 10.7717/peerj.12324 (PMC8684741; doi:10.7717/peerj.12324)
Supplement: Supplemental Information 6 [file peerj-09-12324-s006.docx]

**Table S6. Broader socio-economic factors associated with women’s early marriage in the full sample of uneducated women aged 12-39 years**

|  | **Hypothesis 3: Combined model** | | | | | | | |
| --- | --- | --- | --- | --- | --- | --- | --- | --- |
|  | **Model 1: Marrying <15 years**  *n*=914^1^ *R*^2^ =0.104 | | **Model 2: Marrying <16 years**  *n*=1,499^2^ *R*^2^ =0.097 | | **Model 3: Marrying <17 years**  *n*=1,841^3^ *R*^2^ =0.098 | | **Model 4: Marrying <18 years**  *n*=2,116^4^ *R*^2^ =0.096 | |
|  | **aOR (95% CI)** | ***p-*value** | **aOR (95% CI)** | ***p-*value** | **aOR (95% CI)** | ***p-*value** | **aOR (95% CI)** | ***p-*value** |
| Women’s age (y) | 0.90 (0.86, 0.94) | <0.001 | 0.91 (0.87, 0.94) | <0.001 | 0.89 (0.86, 0.93) | <0.001 | 0.90 (0.86, 0.93) | <0.001 |
| Asset score |  |  |  |  |  |  |  |  |
| Poorest | 0.84 (0.41, 1.71) | 0.623 | 0.81 (0.43, 1.53) | 0.519 | 0.88 (0.48, 1.63) | 0.696 | 0.79 (0.44, 1.44) | 0.446 |
| 2^nd^ poorest | 0.75 (0.37, 1.51) | 0.422 | 0.66 (0.35, 1.24) | 0.200 | 0.71 (0.39, 1.30) | 0.268 | 0.66 (0.37, 1.19) | 0.167 |
| Mid | 0.86 (0.43, 1.71) | 0.659 | 0.79 (0.42, 1.48) | 0.462 | 0.80 (0.44, 1.46) | 0.468 | 0.75 (0.42, 1.35) | 0.337 |
| 2^nd^ richest | 1.60 (0.74, 3.46) | 0.234 | 1.38 (0.68, 2.79) | 0.372 | 1.40 (0.71, 2.77) | 0.334 | 1.39 (0.71, 2.70) | 0.334 |
| Richest (ref) | 1.00 |  | 1.00 |  | 1.00 |  | 1.00 |  |
| Agrarian land |  |  |  |  |  |  |  |  |
| None | 1.51 (0.78, 2.93) | 0.225 | 1.25 (0.70, 2.25) | 0.446 | 1.16 (0.66, 2.04) | 0.600 | 1.28 (0.74, 2.21) | 0.379 |
| 0.01 to 0.5 hectares | 1.62 (0.84, 3.14) | 0.150 | 1.44 (0.80, 2.58) | 0.220 | 1.43 (0.81, 2.52) | 0.212 | 1.65 (0.95, 2.85) | 0.074 |
| 0.51 to 0.99 hectares | 1.31 (0.61, 2.85) | 0.489 | 1.21 (0.61, 2.42) | 0.585 | 1.23 (0.63, 2.39) | 0.512 | 1.37 (0.72, 2.63) | 0.339 |
| ≥1 hectare (ref) | 1.00 |  | 1.00 |  | 1.00 |  | 1.00 |  |
| Access to big bazaar |  |  |  |  |  |  |  |  |
| <30 min (ref) | 1.00 |  | 1.00 |  | 1.00 |  | 1.00 |  |
| 30-59 minutes | 0.93 (0.57, 1.54) | 0.784 | 0.87 (0.55, 1.37) | 0.546 | 0.84 (0.55, 1.30) | 0.442 | 0.86 (0.56, 1.31) | 0.488 |
| 60-89 minutes | 1.11 (0.59, 2.09) | 0.736 | 1.28 (0.72, 2.26) | 0.402 | 1.13 (0.65, 1.95) | 0.666 | 1.11 (0.65, 1.88) | 0.711 |
| ≥90 minutes | 1.33 (0.62, 2.85) | 0.469 | 1.19 (0.57, 2.45) | 0.646 | 1.03 (0.51, 2.05) | 0.937 | 1.04 (0.53, 2.05) | 0.899 |
| Caste |  |  |  |  |  |  |  |  |
| Disadvantaged: Dalit | 1.08 (0.55, 2.14) | 0.820 | 1.00 (0.55, 1.82) | 0.994 | 1.02 (0.57, 1.82) | 0.951 | 1.08 (0.61, 1.91) | 0.790 |
| Disadvantaged: Muslim | 1.04 (0.56, 1.93) | 0.912 | 0.91 (0.52, 1.60) | 0.750 | 0.93 (0.54, 1.59) | 0.785 | 0.92 (0.55, 1.56) | 0.765 |
| Middle: Janjati, Terai castes | 0.98 (0.55, 1.75) | 0.950 | 0.97 (0.58, 1.62) | 0.908 | 0.97 (0.59, 1.60) | 0.908 | 0.93 (0.57, 1.52) | 0.773 |
| Advantaged: Yadav, Brahmin (ref) | 1.00 |  | 1.00 |  | 1.00 |  | 1.00 |  |
| Intercept | 29.88 (6.14, 145.44) | <0.001 | 67.30 (16.19, 279.72) | <0.001 | 115.71 (29.97, 446.80) | <0.001 | 115.51 (31.19, 427.75) | <0.001 |

Models include fixed and random effects estimates for geographic clusters and control for trial arm. aOR, adjusted Odds Ratio. CI, 95% Confidence Interval. ^1^*n*=206 married ≥18y vs *n*=708 married <15y. ^2^*n*=206 married ≥18y vs *n*=1,293 married <16y. ^3^*n*=206 married ≥18y vs *n*=1,635 married <17y. ^4^*n*=206 married ≥18y vs *n*=1,910 married <18y.
