# Supplementary material for: Quantifying the association of natal household wealth with women’s early marriage in Nepal
Source: PeerJ. 2021 Dec 16;9:e12324. doi: 10.7717/peerj.12324 (PMC8684741; doi:10.7717/peerj.12324)
Supplement: Supplemental Information 7 [file peerj-09-12324-s007.docx]

**Table S7. Broader socio-economic factors associated with women being uneducated in the full sample of women aged 12-39 years**

|  | **Hypothesis 4** | | | |
| --- | --- | --- | --- | --- |
|  | **Model 1: Natal household asset score** *n*=3,379^1^ *R*^2^ =0.190 | | **Model 2: Broader socio-economic factors**  *n*=3,379^1^ *R*^2^ =0.347 | |
|  | **OR (95% CI)** | ***p-*value** | **aOR (95% CI)** | ***p-*value** |
| Women’s age (y) | 1.16 (1.13, 1.19) | <0.001 | 1.19 (1.16, 1.22) | <0.001 |
| Asset score | 1.00 |  | 1.00 |  |
| Poorest | 8.99 (6.83, 11.83) | <0.001 | 7.09 (5.19, 9.68) | <0.001 |
| 2^nd^ poorest | 3.53 (2.75, 4.53) | <0.001 | 3.42 (2.59, 4.53) | <0.001 |
| Mid | 3.11 (2.44, 3.96) | <0.001 | 3.27 (2.49, 4.29) | <0.001 |
| 2^nd^ richest | 1.52 (1.19, 1.94) | 0.001 | 1.63 (1.24, 2.14) | <0.001 |
| Richest (ref) |  |  | 1.00 |  |
| Agrarian land |  |  |  |  |
| None |  |  | 2.86 (2.15, 3.80) | <0.001 |
| 0.01 to 0.5 hectares |  |  | 1.79 (1.38, 2.33) | <0.001 |
| 0.51 to 0.99 hectares |  |  | 1.25 (0.94, 1.67) | 0.127 |
| ≥1 hectare (ref) |  |  | 1.00 |  |
| Access to big bazaar |  |  |  |  |
| <30 min (ref) |  |  | 1.00 |  |
| 30-59 minutes |  |  | 1.12 (0.89, 1.40) | 0.329 |
| 60-89 minutes |  |  | 1.10 (0.84, 1.45) | 0.493 |
| ≥90 minutes |  |  | 1.39 (0.97, 1.99) | 0.070 |
| Caste |  |  |  |  |
| Disadvantaged: Dalit |  |  | 1.65 (1.24, 2.19) | 0.001 |
| Disadvantaged: Muslim |  |  | 6.73 (4.91, 9.24) | <0.001 |
| Middle: Janjati, Terai castes |  |  | 0.90 (0.72, 1.12) | 0.347 |
| Advantaged: Yadav, Brahmin (ref) |  |  | 1.00 |  |
| Intercept | 0.04 (0.02, 0.08) | <0.001 | 0.01 (0.00, 0.02) | <0.001 |

Models include fixed and random effects estimates for geographic clusters and control for trial arm. aOR, adjusted Odds Ratio. CI, 95% Confidence Interval. ^1^*n*=1,263 educated (≥1y schooling) vs *n*=2,116 uneducated.
